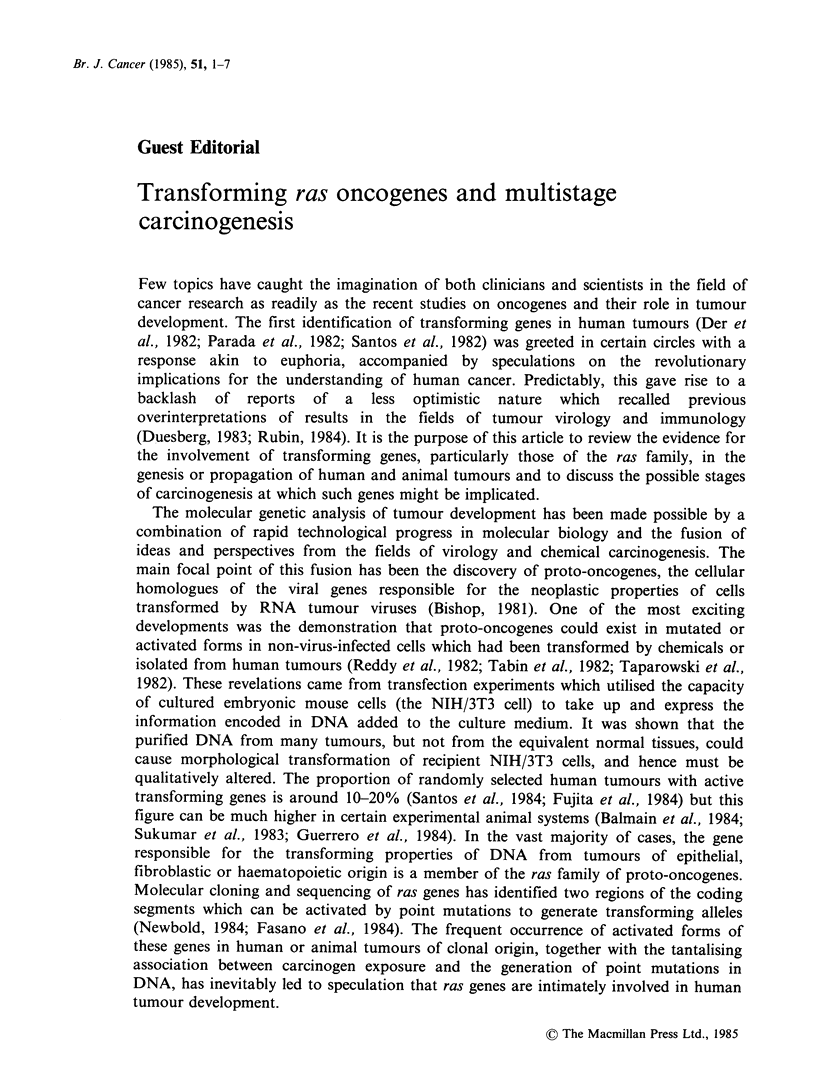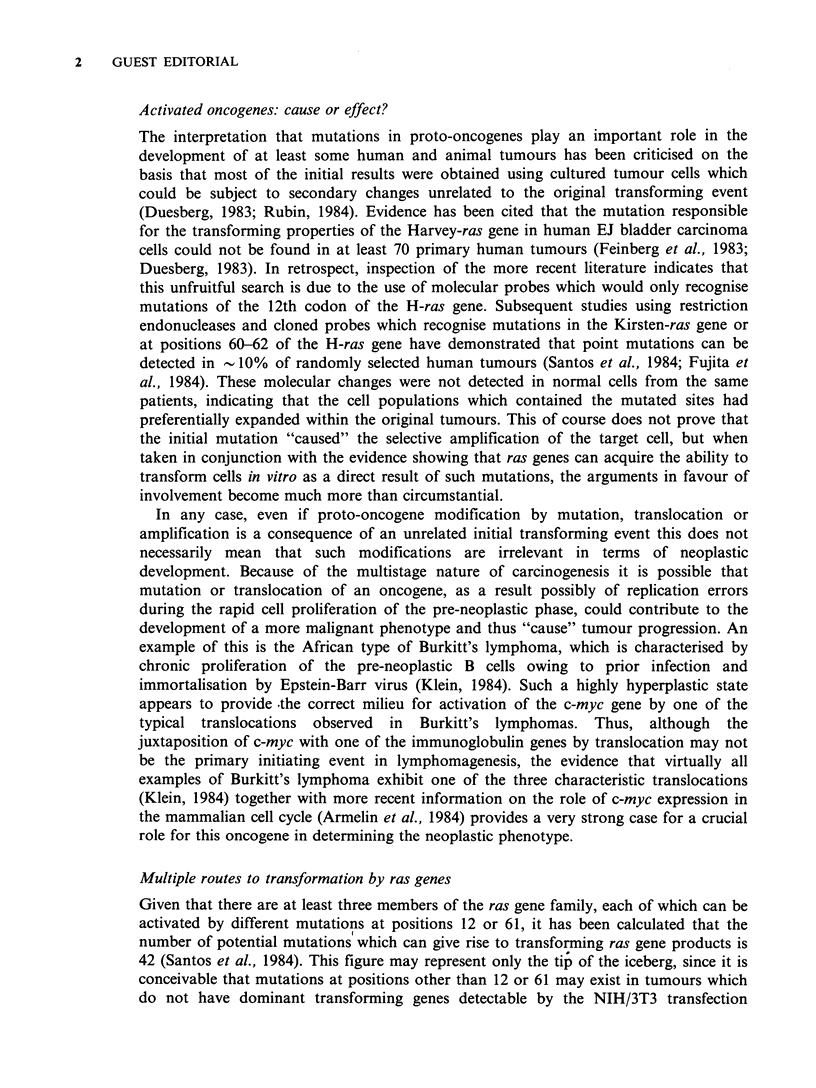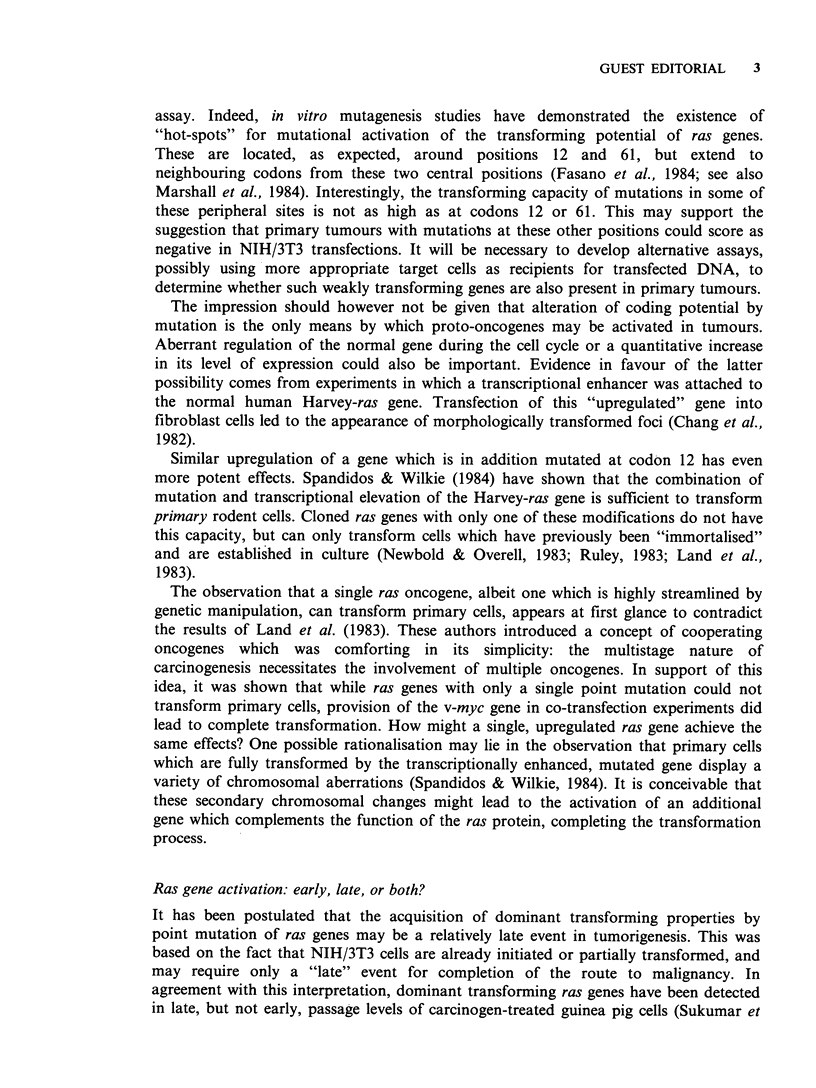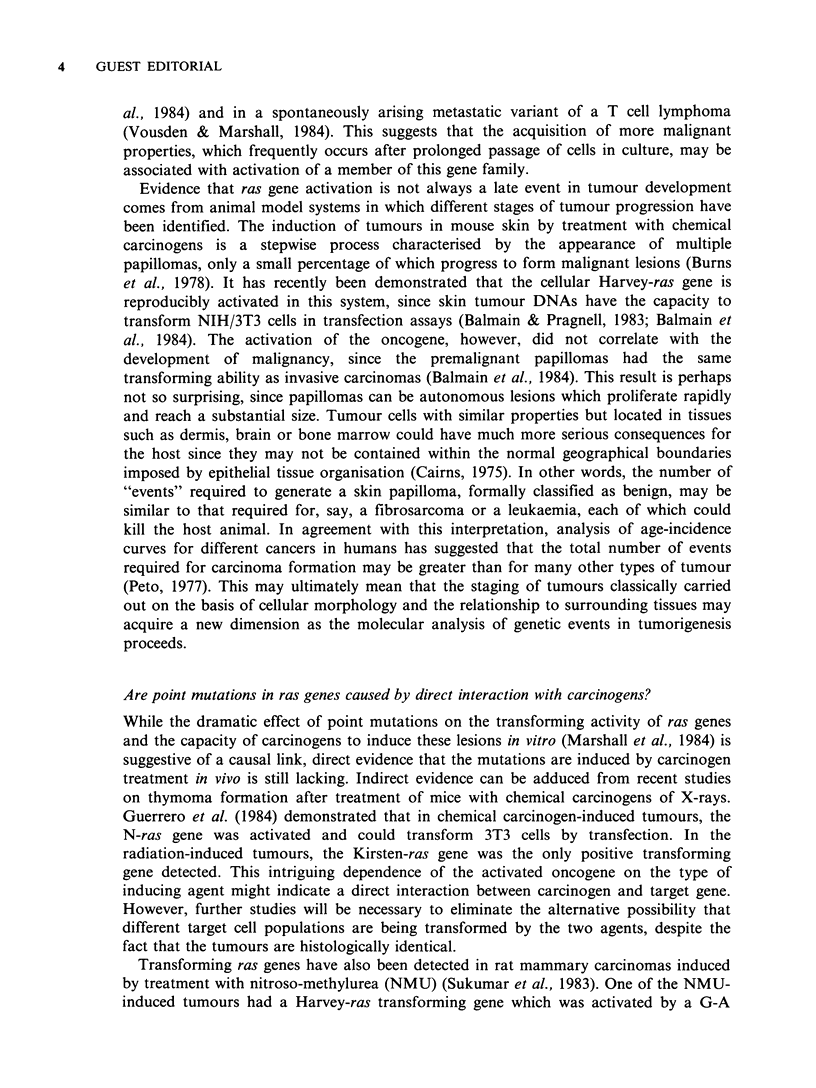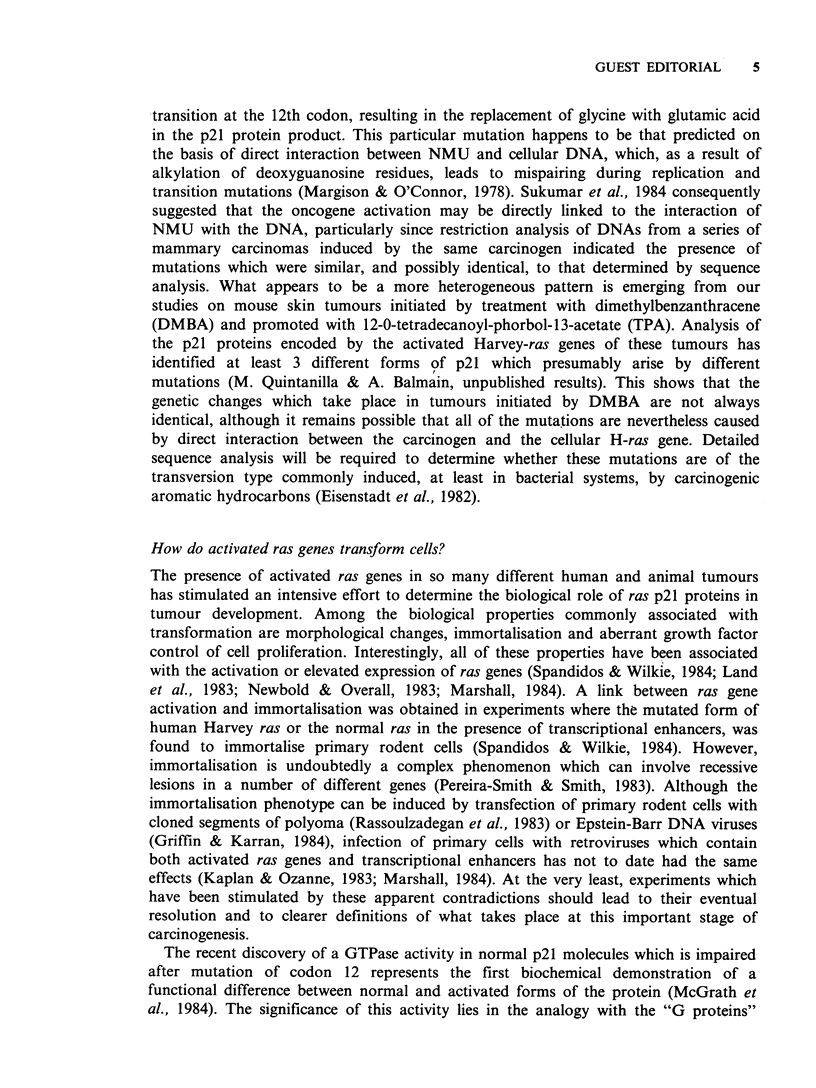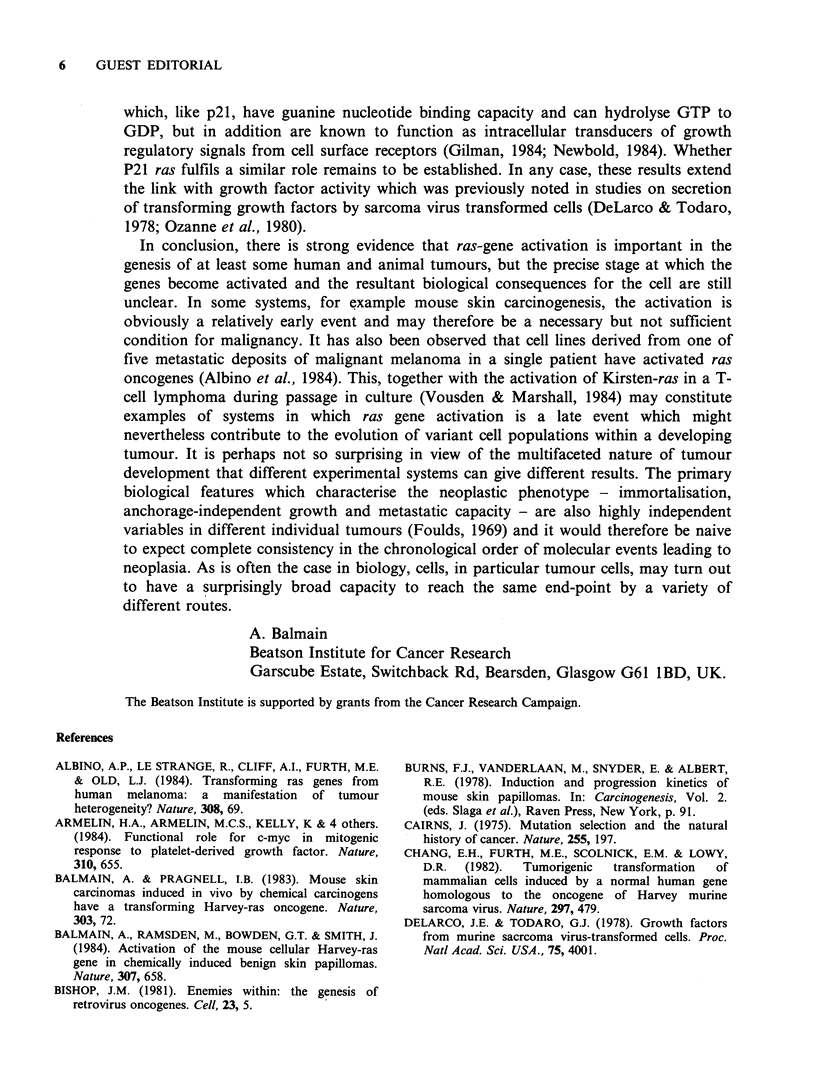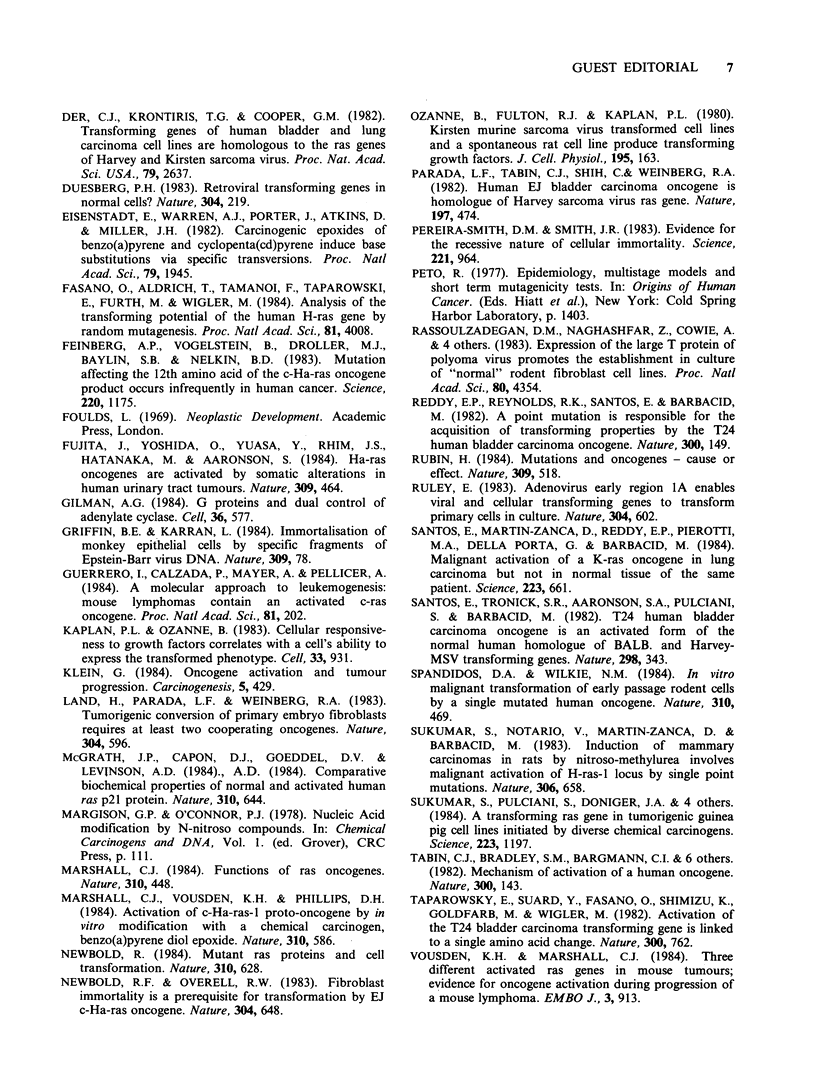# Transforming ras oncogenes and multistage carcinogenesis.

**DOI:** 10.1038/bjc.1985.1

**Published:** 1985-01

**Authors:** A. Balmain


*Br. J. Cancer* (1985), **51**, 1–7

### Guest Editorial

### Transforming *ras* oncogenes and multistage carcinogenesis

Few topics have caught the imagination of both clinicians and scientists in the field of cancer research as readily as the recent studies on oncogenes and their role in tumour development. The first identification of transforming genes in human tumours (Der *et al.*, 1982; Parada *et al.*, 1982; Santos *et al.*, 1982) was greeted in certain circles with a response akin to euphoria, accompanied by speculations on the revolutionary implications for the understanding of human cancer. Predictably, this gave rise to a backlash of reports of a less optimistic nature which recalled previous overinterpretations of results in the fields of tumour virology and immunology (Duesberg, 1983; Rubin, 1984). It is the purpose of this article to review the evidence for the involvement of transforming genes, particularly those of the *ras* family, in the genesis or propagation of human and animal tumours and to discuss the possible stages of carcinogenesis at which such genes might be implicated.

The molecular genetic analysis of tumour development has been made possible by a combination of rapid technological progress in molecular biology and the fusion of ideas and perspectives from the fields of virology and chemical carcinogenesis. The main focal point of this fusion has been the discovery of proto-oncogenes, the cellular homologues of the viral genes responsible for the neoplastic properties of cells transformed by RNA tumour viruses (Bishop, 1981). One of the most exciting developments was the demonstration that proto-oncogenes could exist in mutated or activated forms in non-virus-infected cells which had been transformed by chemicals or isolated from human tumours (Reddy *et al.*, 1982; Tabin *et al.*, 1982; Taparowski *et al.*, 1982). These revelations came from transfection experiments which utilised the capacity of cultured embryonic mouse cells (the NIH/3T3 cell) to take up and express the information encoded in DNA added to the culture medium. It was shown that the purified DNA from many tumours, but not from the equivalent normal tissues, could cause morphological transformation of recipient NIH/3T3 cells, and hence must be qualitatively altered. The proportion of randomly selected human tumours with active transforming genes is around 10–20% (Santos *et al.*, 1984; Fujita *et al.*, 1984) but this figure can be much higher in certain experimental animal systems (Balmain *et al.*, 1984; Sukumar *et al.*, 1983; Guerrero *et al.*, 1984). In the vast majority of cases, the gene responsible for the transforming properties of DNA from tumours of epithelial, fibroblastic or haematopoietic origin is a member of the *ras* family of proto-oncogenes. Molecular cloning and sequencing of *ras* genes has identified two regions of the coding segments which can be activated by point mutations to generate transforming alleles (Newbold, 1984; Fasano *et al.*, 1984). The frequent occurrence of activated forms of these genes in human or animal tumours of clonal origin, together with the tantalising association between carcinogen exposure and the generation of point mutations in DNA, has inevitably led to speculation that *ras* genes are intimately involved in human tumour development.

© The Macmillan Press Ltd., 1985

*Activated oncogenes: cause or effect?*

The interpretation that mutations in proto-oncogenes play an important role in the development of at least some human and animal tumours has been criticised on the basis that most of the initial results were obtained using cultured tumour cells which could be subject to secondary changes unrelated to the original transforming event (Duesberg, 1983; Rubin, 1984). Evidence has been cited that the mutation responsible for the transforming properties of the Harvey-*ras* gene in human EJ bladder carcinoma cells could not be found in at least 70 primary human tumours (Feinberg *et al.,* 1983; Duesberg, 1983). In retrospect, inspection of the more recent literature indicates that this unfruitful search is due to the use of molecular probes which would only recognise mutations of the 12th codon of the H-*ras* gene. Subsequent studies using restriction endonucleases and cloned probes which recognise mutations in the Kirsten-*ras* gene or at positions 60–62 of the H-*ras* gene have demonstrated that point mutations can be detected in ~10% of randomly selected human tumours (Santos *et al.,* 1984; Fujita *et al.,* 1984). These molecular changes were not detected in normal cells from the same patients, indicating that the cell populations which contained the mutated sites had preferentially expanded within the original tumours. This of course does not prove that the initial mutation "caused" the selective amplification of the target cell, but when taken in conjunction with the evidence showing that *ras* genes can acquire the ability to transform cells *in vitro* as a direct result of such mutations, the arguments in favour of involvement become much more than circumstantial.

In any case, even if proto-oncogene modification by mutation, translocation or amplification is a consequence of an unrelated initial transforming event this does not necessarily mean that such modifications are irrelevant in terms of neoplastic development. Because of the multistage nature of carcinogenesis it is possible that mutation or translocation of an oncogene, as a result possibly of replication errors during the rapid cell proliferation of the pre-neoplastic phase, could contribute to the development of a more malignant phenotype and thus "cause" tumour progression. An example of this is the African type of Burkitt's lymphoma, which is characterised by chronic proliferation of the pre-neoplastic B cells owing to prior infection and immortalisation by Epstein-Barr virus (Klein, 1984). Such a highly hyperplastic state appears to provide the correct milieu for activation of the c-*myc* gene by one of the typical translocations observed in Burkitt's lymphomas. Thus, although the juxtaposition of c-*myc* with one of the immunoglobulin genes by translocation may not be the primary initiating event in lymphomagenesis, the evidence that virtually all examples of Burkitt's lymphoma exhibit one of the three characteristic translocations (Klein, 1984) together with more recent information on the role of c-*myc* expression in the mammalian cell cycle (Armelin *et al.,* 1984) provides a very strong case for a crucial role for this oncogene in determining the neoplastic phenotype.

*Multiple routes to transformation by ras genes*

Given that there are at least three members of the *ras* gene family, each of which can be activated by different mutations at positions 12 or 61, it has been calculated that the number of potential mutations which can give rise to transforming *ras* gene products is 42 (Santos *et al.,* 1984). This figure may represent only the tip of the iceberg, since it is conceivable that mutations at positions other than 12 or 61 may exist in tumours which do not have dominant transforming genes detectable by the NIH/3T3 transfection assay. Indeed, *in vitro* mutagenesis studies have demonstrated the existence of "hot-spots" for mutational activation of the transforming potential of *ras* genes. These are located, as expected, around positions 12 and 61, but extend to neighbouring codons from these two central positions (Fasano *et al.,* 1984; see also Marshall *et al.,* 1984). Interestingly, the transforming capacity of mutations in some of these peripheral sites is not as high as at codons 12 or 61. This may support the suggestion that primary tumours with mutations at these other positions could score as negative in NIH/3T3 transfections. It will be necessary to develop alternative assays, possibly using more appropriate target cells as recipients for transfected DNA, to determine whether such weakly transforming genes are also present in primary tumours.

The impression should however not be given that alteration of coding potential by mutation is the only means by which proto-oncogenes may be activated in tumours. Aberrant regulation of the normal gene during the cell cycle or a quantitative increase in its level of expression could also be important. Evidence in favour of the latter possibility comes from experiments in which a transcriptional enhancer was attached to the normal human Harvey-*ras* gene. Transfection of this "upregulated" gene into fibroblast cells led to the appearance of morphologically transformed foci (Chang *et al.,* 1982).

Similar upregulation of a gene which is in addition mutated at codon 12 has even more potent effects. Spandidos & Wilkie (1984) have shown that the combination of mutation and transcriptional elevation of the Harvey-*ras* gene is sufficient to transform *primary* rodent cells. Cloned *ras* genes with only one of these modifications do not have this capacity, but can only transform cells which have previously been "immortalised" and are established in culture (Newbold & Overell, 1983; Ruley, 1983; Land *et al.,* 1983).

The observation that a single *ras* oncogene, albeit one which is highly streamlined by genetic manipulation, can transform primary cells, appears at first glance to contradict the results of Land *et al.* (1983). These authors introduced a concept of cooperating oncogenes which was comforting in its simplicity: the multistage nature of carcinogenesis necessitates the involvement of multiple oncogenes. In support of this idea, it was shown that while *ras* genes with only a single point mutation could not transform primary cells, provision of the v-*myc* gene in co-transfection experiments did lead to complete transformation. How might a single, upregulated *ras* gene achieve the same effects? One possible rationalisation may lie in the observation that primary cells which are fully transformed by the transcriptionally enhanced, mutated gene display a variety of chromosomal aberrations (Spandidos & Wilkie, 1984). It is conceivable that these secondary chromosomal changes might lead to the activation of an additional gene which complements the function of the *ras* protein, completing the transformation process.

### *Ras gene activation: early, late, or both?*

It has been postulated that the acquisition of dominant transforming properties by point mutation of *ras* genes may be a relatively late event in tumorigenesis. This was based on the fact that NIH/3T3 cells are already initiated or partially transformed, and may require only a "late" event for completion of the route to malignancy. In agreement with this interpretation, dominant transforming *ras* genes have been detected in late, but not early, passage levels of carcinogen-treated guinea pig cells (Sukumar *et*

*al.*, 1984) and in a spontaneously arising metastatic variant of a T cell lymphoma (Vousden & Marshall, 1984). This suggests that the acquisition of more malignant properties, which frequently occurs after prolonged passage of cells in culture, may be associated with activation of a member of this gene family.

Evidence that *ras* gene activation is not always a late event in tumour development comes from animal model systems in which different stages of tumour progression have been identified. The induction of tumours in mouse skin by treatment with chemical carcinogens is a stepwise process characterised by the appearance of multiple papillomas, only a small percentage of which progress to form malignant lesions (Burns *et al.*, 1978). It has recently been demonstrated that the cellular Harvey-*ras* gene is reproducibly activated in this system, since skin tumour DNAs have the capacity to transform NIH/3T3 cells in transfection assays (Balmain & Pragnell, 1983; Balmain *et al.*, 1984). The activation of the oncogene, however, did not correlate with the development of malignancy, since the premalignant papillomas had the same transforming ability as invasive carcinomas (Balmain *et al.*, 1984). This result is perhaps not so surprising, since papillomas can be autonomous lesions which proliferate rapidly and reach a substantial size. Tumour cells with similar properties but located in tissues such as dermis, brain or bone marrow could have much more serious consequences for the host since they may not be contained within the normal geographical boundaries imposed by epithelial tissue organisation (Cairns, 1975). In other words, the number of "events" required to generate a skin papilloma, formally classified as benign, may be similar to that required for, say, a fibrosarcoma or a leukaemia, each of which could kill the host animal. In agreement with this interpretation, analysis of age-incidence curves for different cancers in humans has suggested that the total number of events required for carcinoma formation may be greater than for many other types of tumour (Peto, 1977). This may ultimately mean that the staging of tumours classically carried out on the basis of cellular morphology and the relationship to surrounding tissues may acquire a new dimension as the molecular analysis of genetic events in tumorigenesis proceeds.

### *Are point mutations in ras genes caused by direct interaction with carcinogens?*

While the dramatic effect of point mutations on the transforming activity of *ras* genes and the capacity of carcinogens to induce these lesions *in vitro* (Marshall *et al.*, 1984) is suggestive of a causal link, direct evidence that the mutations are induced by carcinogen treatment *in vivo* is still lacking. Indirect evidence can be adduced from recent studies on thymoma formation after treatment of mice with chemical carcinogens of X-rays. Guerrero *et al.* (1984) demonstrated that in chemical carcinogen-induced tumours, the N-*ras* gene was activated and could transform 3T3 cells by transfection. In the radiation-induced tumours, the Kirsten-*ras* gene was the only positive transforming gene detected. This intriguing dependence of the activated oncogene on the type of inducing agent might indicate a direct interaction between carcinogen and target gene. However, further studies will be necessary to eliminate the alternative possibility that different target cell populations are being transformed by the two agents, despite the fact that the tumours are histologically identical.

Transforming *ras* genes have also been detected in rat mammary carcinomas induced by treatment with nitroso-methylurea (NMU) (Sukumar *et al.*, 1983). One of the NMU-induced tumours had a Harvey-*ras* transforming gene which was activated by a G-A

transition at the 12th codon, resulting in the replacement of glycine with glutamic acid in the p21 protein product. This particular mutation happens to be that predicted on the basis of direct interaction between NMU and cellular DNA, which, as a result of alkylation of deoxyguanosine residues, leads to mispairing during replication and transition mutations (Margison & O'Connor, 1978). Sukumar *et al.*, 1984 consequently suggested that the oncogene activation may be directly linked to the interaction of NMU with the DNA, particularly since restriction analysis of DNAs from a series of mammary carcinomas induced by the same carcinogen indicated the presence of mutations which were similar, and possibly identical, to that determined by sequence analysis. What appears to be a more heterogeneous pattern is emerging from our studies on mouse skin tumours initiated by treatment with dimethylbenzanthracene (DMBA) and promoted with 12-0-tetradecanoyl-phorbol-13-acetate (TPA). Analysis of the p21 proteins encoded by the activated Harvey-*ras* genes of these tumours has identified at least 3 different forms of p21 which presumably arise by different mutations (M. Quintanilla & A. Balmain, unpublished results). This shows that the genetic changes which take place in tumours initiated by DMBA are not always identical, although it remains possible that all of the mutations are nevertheless caused by direct interaction between the carcinogen and the cellular H-*ras* gene. Detailed sequence analysis will be required to determine whether these mutations are of the transversion type commonly induced, at least in bacterial systems, by carcinogenic aromatic hydrocarbons (Eisenstadt *et al.*, 1982).

*How do activated ras genes transform cells?*

The presence of activated *ras* genes in so many different human and animal tumours has stimulated an intensive effort to determine the biological role of *ras* p21 proteins in tumour development. Among the biological properties commonly associated with transformation are morphological changes, immortalisation and aberrant growth factor control of cell proliferation. Interestingly, all of these properties have been associated with the activation or elevated expression of *ras* genes (Spandidos & Wilkie, 1984; Land *et al.*, 1983; Newbold & Overall, 1983; Marshall, 1984). A link between *ras* gene activation and immortalisation was obtained in experiments where the mutated form of human Harvey *ras* or the normal *ras* in the presence of transcriptional enhancers, was found to immortalise primary rodent cells (Spandidos & Wilkie, 1984). However, immortalisation is undoubtedly a complex phenomenon which can involve recessive lesions in a number of different genes (Pereira-Smith & Smith, 1983). Although the immortalisation phenotype can be induced by transfection of primary rodent cells with cloned segments of polyoma (Rassoulzadegan *et al.*, 1983) or Epstein-Barr DNA viruses (Griffin & Karran, 1984), infection of primary cells with retroviruses which contain both activated *ras* genes and transcriptional enhancers has not to date had the same effects (Kaplan & Ozanne, 1983; Marshall, 1984). At the very least, experiments which have been stimulated by these apparent contradictions should lead to their eventual resolution and to clearer definitions of what takes place at this important stage of carcinogenesis.

The recent discovery of a GTPase activity in normal p21 molecules which is impaired after mutation of codon 12 represents the first biochemical demonstration of a functional difference between normal and activated forms of the protein (McGrath *et al.*, 1984). The significance of this activity lies in the analogy with the "G proteins"

which, like p21, have guanine nucleotide binding capacity and can hydrolyse GTP to GDP, but in addition are known to function as intracellular transducers of growth regulatory signals from cell surface receptors (Gilman, 1984; Newbold, 1984). Whether P21 *ras* fulfils a similar role remains to be established. In any case, these results extend the link with growth factor activity which was previously noted in studies on secretion of transforming growth factors by sarcoma virus transformed cells (DeLarco & Todaro, 1978; Ozanne *et al.,* 1980).

In conclusion, there is strong evidence that *ras*-gene activation is important in the genesis of at least some human and animal tumours, but the precise stage at which the genes become activated and the resultant biological consequences for the cell are still unclear. In some systems, for example mouse skin carcinogenesis, the activation is obviously a relatively early event and may therefore be a necessary but not sufficient condition for malignancy. It has also been observed that cell lines derived from one of five metastatic deposits of malignant melanoma in a single patient have activated *ras* oncogenes (Albino *et al.,* 1984). This, together with the activation of Kirsten-*ras* in a T-cell lymphoma during passage in culture (Vousden & Marshall, 1984) may constitute examples of systems in which *ras* gene activation is a late event which might nevertheless contribute to the evolution of variant cell populations within a developing tumour. It is perhaps not so surprising in view of the multifaceted nature of tumour development that different experimental systems can give different results. The primary biological features which characterise the neoplastic phenotype – immortalisation, anchorage-independent growth and metastatic capacity – are also highly independent variables in different individual tumours (Foulds, 1969) and it would therefore be naive to expect complete consistency in the chronological order of molecular events leading to neoplasia. As is often the case in biology, cells, in particular tumour cells, may turn out to have a surprisingly broad capacity to reach the same end-point by a variety of different routes.

A. Balmain
Beatson Institute for Cancer Research
Garscube Estate, Switchback Rd, Bearsden, Glasgow G61 1BD, UK.

The Beatson Institute is supported by grants from the Cancer Research Campaign.

**References**

ALBINO, A.P., LE STRANGE, R., CLIFF, A.I., FURTH, M.E. & OLD, L.J. (1984). Transforming ras genes from human melanoma: a manifestation of tumour heterogeneity? *Nature,* **308,** 69.

ARMELIN, H.A., ARMELIN, M.C.S., KELLY, K & 4 others. (1984). Functional role for c-myc in mitogenic response to platelet-derived growth factor. *Nature,* **310,** 655.

BALMAIN, A. & PRAGNELL, I.B. (1983). Mouse skin carcinomas induced in vivo by chemical carcinogens have a transforming Harvey-ras oncogene. *Nature,* **303,** 72.

BALMAIN, A., RAMSDEN, M., BOWDEN, G.T. & SMITH, J. (1984). Activation of the mouse cellular Harvey-ras gene in chemically induced benign skin papillomas. *Nature,* **307,** 658.

BISHOP, J.M. (1981). Enemies within: the genesis of retrovirus oncogenes. *Cell,* **23,** 5.

BURNS, F.J., VANDERLAAN, M., SNYDER, E. & ALBERT, R.E. (1978). Induction and progression kinetics of mouse skin papillomas. In: *Carcinogenesis,* Vol. 2. (eds. Slaga *et al.*), Raven Press, New York, p. 91.

CAIRNS, J. (1975). Mutation selection and the natural history of cancer. *Nature,* **255,** 197.

CHANG, E.H., FURTH, M.E., SCOLNICK, E.M. & LOWY, D.R. (1982). Tumorigenic transformation of mammalian cells induced by a normal human gene homologous to the oncogene of Harvey murine sarcoma virus. *Nature,* **297,** 479.

DELARCO, J.E. & TODARO, G.J. (1978). Growth factors from murine sacrcoma virus-transformed cells. *Proc. Natl Acad. Sci. USA.,* **75,** 4001.

DER, C.J., KRONTIRIS, T.G. & COOPER, G.M. (1982). Transforming genes of human bladder and lung carcinoma cell lines are homologous to the ras genes of Harvey and Kirsten sarcoma virus. *Proc. Nat. Acad. Sci. USA.*, **79**, 2637.

DUESBERG, P.H. (1983). Retroviral transforming genes in normal cells? *Nature*, **304**, 219.

EISENSTADT, E., WARREN, A.J., PORTER, J., ATKINS, D. & MILLER, J.H. (1982). Carcinogenic epoxides of benzo(a)pyrene and cyclopenta(cd)pyrene induce base substitutions via specific transversions. *Proc. Natl Acad. Sci.*, **79**, 1945.

FASANO, O., ALDRICH, T., TAMANOI, F., TAPAROWSKI, E., FURTH, M. & WIGLER, M. (1984). Analysis of the transforming potential of the human H-ras gene by random mutagenesis. *Proc. Natl Acad. Sci.*, **81**, 4008.

FEINBERG, A.P., VOGELSTEIN, B., DROLLER, M.J., BAYLIN, S.B. & NELKIN, B.D. (1983). Mutation affecting the 12th amino acid of the c-Ha-ras oncogene product occurs infrequently in human cancer. *Science*, **220**, 1175.

FOULDS, L. (1969). *Neoplastic Development*. Academic Press, London.

FUJITA, J., YOSHIDA, O., YUASA, Y., RHIM, J.S., HATANAKA, M. & AARONSON, S. (1984). Ha-ras oncogenes are activated by somatic alterations in human urinary tract tumours. *Nature*, **309**, 464.

GILMAN, A.G. (1984). G proteins and dual control of adenylate cyclase. *Cell*, **36**, 577.

GRIFFIN, B.E. & KARRAN, L. (1984). Immortalisation of monkey epithelial cells by specific fragments of Epstein-Barr virus DNA. *Nature*, **309**, 78.

GUERRERO, I., CALZADA, P., MAYER, A. & PELLICER, A. (1984). A molecular approach to leukemogenesis: mouse lymphomas contain an activated c-ras oncogene. *Proc. Natl Acad. Sci.*, **81**, 202.

KAPLAN, P.L. & OZANNE, B. (1983). Cellular responsiveness to growth factors correlates with a cell's ability to express the transformed phenotype. *Cell*, **33**, 931.

KLEIN, G. (1984). Oncogene activation and tumour progression. *Carcinogenesis*, **5**, 429.

LAND, H., PARADA, L.F. & WEINBERG, R.A. (1983). Tumorigenic conversion of primary embryo fibroblasts requires at least two cooperating oncogenes. *Nature*, **304**, 596.

McGRATH, J.P., CAPON, D.J., GOEDDEL, D.V. & LEVINSON, A.D. (1984)., A.D. (1984). Comparative biochemical properties of normal and activated human *ras* p21 protein. *Nature*, **310**, 644.

MARGISON, G.P. & O'CONNOR, P.J. (1978). Nucleic Acid modification by N-nitroso compounds. In: *Chemical Carcinogens and DNA*, Vol. 1. (ed. Grover), CRC Press, p. 111.

MARSHALL, C.J. (1984). Functions of ras oncogenes. *Nature*, **310**, 448.

MARSHALL, C.J., VOUSDEN, K.H. & PHILLIPS, D.H. (1984). Activation of c-Ha-ras-1 proto-oncogene by *in vitro* modification with a chemical carcinogen, benzo(a)pyrene diol epoxide. *Nature*, **310**, 586.

NEWBOLD, R. (1984). Mutant ras proteins and cell transformation. *Nature*, **310**, 628.

NEWBOLD, R.F. & OVERELL, R.W. (1983). Fibroblast immortality is a prerequisite for transformation by EJ c-Ha-ras oncogene. *Nature*, **304**, 648.

OZANNE, B., FULTON, R.J. & KAPLAN, P.L. (1980). Kirsten murine sarcoma virus transformed cell lines and a spontaneous rat cell line produce transforming growth factors. *J. Cell. Physiol.*, **195**, 163.

PARADA, L.F., TABIN, C.J., SHIH, C.& WEINBERG, R.A. (1982). Human EJ bladder carcinoma oncogene is homologue of Harvey sarcoma virus ras gene. *Nature*, **197**, 474.

PEREIRA-SMITH, D.M. & SMITH, J.R. (1983). Evidence for the recessive nature of cellular immortality. *Science*, **221**, 964.

PETO, R. (1977). Epidemiology, multistage models and short term mutagenicity tests. In: *Origins of Human Cancer*. (Eds. Hiatt *et al.*), New York: Cold Spring Harbor Laboratory, p. 1403.

RASSOULZADEGAN, D.M., NAGHASHFAR, Z., COWIE, A. & 4 others. (1983). Expression of the large T protein of polyoma virus promotes the establishment in culture of "normal" rodent fibroblast cell lines. *Proc. Natl Acad. Sci.*, **80**, 4354.

REDDY, E.P., REYNOLDS, R.K., SANTOS, E. & BARBACID, M. (1982). A point mutation is responsible for the acquisition of transforming properties by the T24 human bladder carcinoma oncogene. *Nature*, **300**, 149.

RUBIN, H. (1984). Mutations and oncogenes – cause or effect. *Nature*, **309**, 518.

RULEY, E. (1983). Adenovirus early region 1A enables viral and cellular transforming genes to transform primary cells in culture. *Nature*, **304**, 602.

SANTOS, E., MARTIN-ZANCA, D., REDDY, E.P., PIEROTTI, M.A., DELLA PORTA, G. & BARBACID, M. (1984). Malignant activation of a K-ras oncogene in lung carcinoma but not in normal tissue of the same patient. *Science*, **223**, 661.

SANTOS, E., TRONICK, S.R., AARONSON, S.A., PULCIANI, S. & BARBACID, M. (1982). T24 human bladder carcinoma oncogene is an activated form of the normal human homologue of BALB. and Harvey-MSV transforming genes. *Nature*, **298**, 343.

SPANDIDOS, D.A. & WILKIE, N.M. (1984). *In vitro* malignant transformation of early passage rodent cells by a single mutated human oncogene. *Nature*, **310**, 469.

SUKUMAR, S., NOTARIO, V., MARTIN-ZANCA, D. & BARBACID, M. (1983). Induction of mammary carcinomas in rats by nitroso-methylurea involves malignant activation of H-ras-1 locus by single point mutations. *Nature*, **306**, 658.

SUKUMAR, S., PULCIANI, S., DONIGER, J.A. & 4 others. (1984). A transforming ras gene in tumorigenic guinea pig cell lines initiated by diverse chemical carcinogens. *Science*, **223**, 1197.

TABIN, C.J., BRADLEY, S.M., BARGMANN, C.I. & 6 others. (1982). Mechanism of activation of a human oncogene. *Nature*, **300**, 143.

TAPAROWSKY, E., SUARD, Y., FASANO, O., SHIMIZU, K., GOLDFARB, M. & WIGLER, M. (1982). Activation of the T24 bladder carcinoma transforming gene is linked to a single amino acid change. *Nature*, **300**, 762.

VOUSDEN, K.H. & MARSHALL, C.J. (1984). Three different activated ras genes in mouse tumours; evidence for oncogene activation during progression of a mouse lymphoma. *EMBO J.*, **3**, 913.